# Genetic diversity in global populations of the critically endangered addax (*Addax nasomaculatus*) and its implications for conservation

**DOI:** 10.1111/eva.13515

**Published:** 2022-12-21

**Authors:** Kara L. Dicks, Alex D. Ball, Lisa Banfield, Violeta Barrios, Mohamed Boufaroua, Abdelkader Chetoui, Justin Chuven, Mark Craig, Mohammed Yousef Al Faqeer, Hamissou Halilou Mallam Garba, Hela Guedara, Abdoulaye Harouna, Jamie Ivy, Chawki Najjar, Marie Petretto, Ricardo Pusey, Thomas Rabeil, Philip Riordan, Helen V. Senn, Ezzedine Taghouti, Tim Wacher, Tim Woodfine, Tania Gilbert

**Affiliations:** ^1^ RZSS WildGenes, Royal Zoological Society of Scotland Edinburgh UK; ^2^ Life Sciences Department Al Ain Zoo Al Ain United Arab Emirates; ^3^ SaharaConservation Saint Maur des Fossés France; ^4^ Direction Générale des Forêts Tunis Tunisia; ^5^ Conservation Biology, Marwell Wildlife Winchester UK; ^6^ Terrestrial & Marine Biodiversity Management Sector, Environment Agency – Abu Dhabi Abu Dhabi United Arab Emirates; ^7^ Ministère de l'Environnement et de la Lutte Contre la Désertification Niamey Niger; ^8^ Noé au Niger Réserve Naturelle Nationale de Termit et Tin‐Toumma Niger; ^9^ San Diego Zoo Wildlife Alliance San Diego California USA; ^10^ Association Tunisienne de la Vie Sauvage Tunis Tunisia; ^11^ School of Biological Sciences, Faculty of Environmental and Life Sciences University of Southampton Southampton UK; ^12^ Conservation & Policy, Zoological Society of London London UK

**Keywords:** *Addax nasomaculatus*, captive populations, conservation genetics, reintroduction, Sahelo‐Saharan antelope, ungulate conservation

## Abstract

Threatened species are frequently patchily distributed across small wild populations, ex situ populations managed with varying levels of intensity and reintroduced populations. Best practice advocates for integrated management across in situ and ex situ populations. Wild addax (*Addax nasomaculatus*) now number fewer than 100 individuals, yet 1000 of addax remain in ex situ populations, which can provide addax for reintroductions, as has been the case in Tunisia since the mid‐1980s. However, integrated management requires genetic data to ascertain the relationships between wild and ex situ populations that have incomplete knowledge of founder origins, management histories, and pedigrees. We undertook a global assessment of genetic diversity across wild, ex situ and reintroduced populations in Tunisia to assist conservation planning for this Critically Endangered species. We show that the remnant wild populations retain more mitochondrial haplotypes that are more diverse than the entirety of the ex situ populations across Europe, North America and the United Arab Emirates, and the reintroduced Tunisian population. Additionally, 1704 SNPs revealed that whilst population structure within the ex situ population is minimal, each population carries unique diversity. Finally, we show that careful selection of founders and subsequent genetic management is vital to ensure genetic diversity is provided to, and minimize drift and inbreeding within reintroductions. Our results highlight a vital need to conserve the last remaining wild addax population, and we provide a genetic foundation for determining integrated conservation strategies to prevent extinction and optimize future reintroductions.

## 1 INTRODUCTION

Genetic diversity is one of three fundamental components of biodiversity in the Convention on Biological Diversity (Laikre et al., 2010; Secretary of the Convention on Biological Diversity, 1992) and the post‐2020 global biodiversity framework (Convention on Biological Diversity, 2020; Hoban et al., 2020). It provides the foundation for evolution, enabling species to adapt to changing environmental and disease conditions (Allendorf, 1986; Frankham et al., 2010) and is therefore essential to the long‐term persistence of species and populations (Lachapelle et al., 2017; Lande & Shannon, 1996; Willi & Hoffmann, 2009). Small, fragmented populations are intrinsically vulnerable to reduced genetic diversity through inbreeding and genetic drift and thus at risk of accumulating deleterious mutations and suffering reduced fitness (Crnokrak & Roff, 1999; Frankham, 1995; Lande, 1994; Lande & Shannon, 1996; Spielman et al., 2004).

Ex situ populations, those managed under intensive human care, can play an essential role in preventing extinction (McGowan et al., 2017), and best practice emphasizes the benefits of developing integrated management plans for threatened species that cover all ex situ and wild in situ populations, including those reintroduced to their indigenous range (Traylor‐Holzer et al., 2019). Management varies substantially amongst ex situ populations, from internationally co‐ordinated pedigree‐based population management programmes that aim to maximize retention of genetic diversity (Ballou & Lacy, 1995), to low‐intensity management of large, independent populations (Wildt et al., 2019), with levels of genetic diversity varying substantially between such populations (Gooley et al., 2020, 2022; Humble et al., 2020; Ogden et al., 2020). Management plans are increasingly incorporating populations with varied management histories, often requiring genetic data to determine the values of different populations relative to each other (Hogg et al., 2017; Marshall et al., 1999; Ogden et al., 2020) and providing information on how they should be managed to achieve a joint goal of species recovery (Hoffmann et al., 2015). This is especially important for species that have substantially larger populations under low‐intensity management than under high‐intensity management or in the wild. Large populations under low‐intensity management may be at greater risk of losing genetic diversity through genetic drift and inbreeding than small populations that are managed intensively to minimize these (Ballou & Lacy, 1995), and species with highly skewed mating systems such as antelope, are at particular risk, as found for scimitar‐horned oryx ex situ populations (*Oryx dammah*, Ogden et al., 2020). In practice, a balance may need to be struck between the availability of individuals for reintroduction, demographic objectives and genetic quality.

**FIGURE 1:**
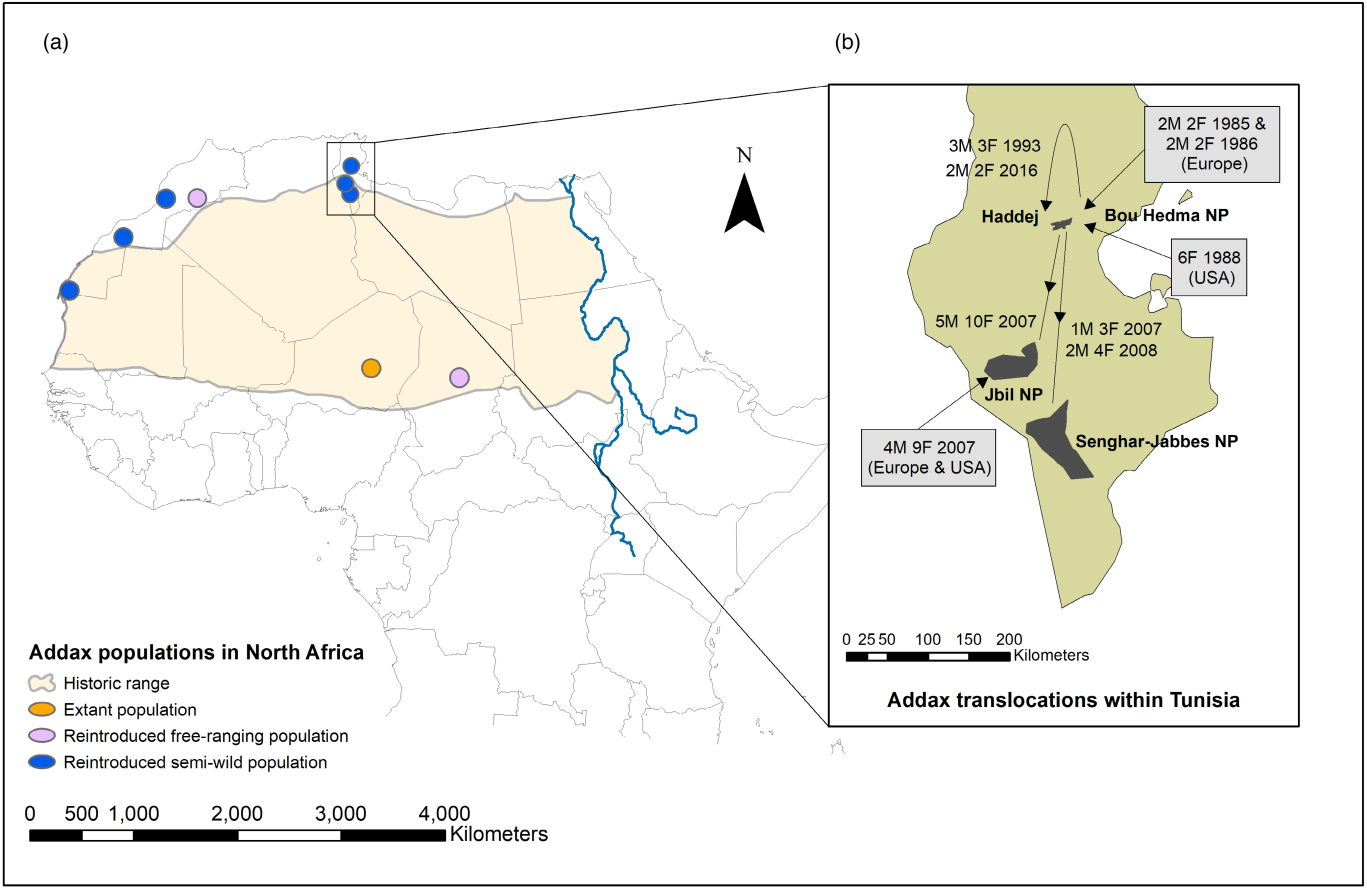
Historical range of addax (a) showing the historic addax range with locations of the remaining population in the Tin Toumma region, Niger and free‐ranging and semiwild reintroduced populations. Inset (b) illustrates the reintroduction history of addax in Tunisia, indicating numbers of males (M) and females (F) translocated from each source population.

The biodiversity of the Sahelo‐Saharan region has suffered a catastrophic decline in megafauna (Brito et al., 2014; Durant et al., 2014), including the Critically Endangered antelope, the addax (*Addax nasomaculatus*), which is now close to extinction in the wild (Durant et al., 2014; IUCN SSC Antelope Specialist Group, 2020; Stabach et al., 2017). Once abundant and widespread in the Sahelo‐Saharan region (Figure 1a), it suffered rapid and catastrophic declines due to hunting, habitat degradation, regional insecurity and, most recently, the impacts of oil exploration in its remaining habitat (IUCN SSC Antelope Specialist Group, 2016). The wild population now numbers <100, restricted to just 0.68% of its historical range (IUCN SSC Antelope Specialist Group, 2020) in one population in the Tin Toumma desert of Niger up to the border with Chad (IUCN SSC Antelope Specialist Group, 2016, 2020).

Whilst addax has been driven to near‐extinction in the wild, the species has been maintained in global zoological institutions since 1920 (Krause, 2016). At the time of writing, there are nearly 1200 ex situ addax registered on the global ZIMS database (Species360, 2022) and several thousand unregistered addax in private populations in the USA and the Arabian Peninsula (IUCN SSC Antelope Specialist Group, 2016; Mallon & Chardonnet, 2020). The three largest regional populations registered in ZIMS are located in (i) the United Arab Emirates (*N* = 556), predominantly in the Al Ain Zoo (AAZ) (*N* = 286) and Environment Agency—Abu Dhabi (EAD) facilities (*N* = 142), (ii) North America (*N* = 256) and (iii) Europe (*N* = 212). The European and North American populations are managed within their respective co‐ordinated population management programmes, the European Association of Zoos and Aquaria's EAZA Ex situ Programme (EEP) and the Species Survival Plan® (SSP) under the Association of Zoos and Aquariums (AZA). As in other species, founder histories are poorly known but founder numbers are thought to be relatively low for addax; the European studbook has 15 wild‐caught founders listed (Krause, 2016) and the international studbook (incorporating the EEP and SSP) indicates up to a maximum of 42 founders (Enright, 2019), but it is likely to be far fewer as missing ancestries and incomplete transaction records may artificially inflate the number of founders recorded. Pedigrees and movements are poorly documented for most addax populations outside these intensively managed programmes. Whilst anecdotal evidence hints towards the existence of novel founders, unmanaged populations in North America and the UAE are probably descended from many of the same wild‐caught individuals as the EEP and SSP populations. The escalating biodiversity crisis in the Sahelo‐Saharan region emphasizes the importance of the ex situ addax population for conservation (Payne & Bro‐Jørgensen, 2016), but a clear understanding of the genetic diversity and structure within the global addax population is crucial for formulating effective conservation action.

Relatively little is known about genetic diversity in either wild or ex situ populations of addax. A recent study by Hempel et al. (2021) generated whole‐genome sequencing data for an ex situ female and mitogenomes from 10 museum samples (1821–1926) originating from across the species' historical range. These data indicated that, prior to recent declines in population numbers, there were relatively low levels of genetic diversity and minimal genetic structure, indicative of a large, highly panmictic population. Previous molecular genetic studies of ex situ addax populations are restricted to either single institutions (Armstrong et al., 2011), region (North America—Spevak et al., 1993) or have focussed on relatedness estimators within a population (North America—Hauser et al., 2022; Ivy et al., 2016), thus representing only a small proportion of the global population. Importantly, different methodologies are used across all studies, prohibiting comparison amongst populations.

Despite the lack of genetic information, ex situ populations have provided a source of animals for a series of historical conservation translocations to Tunisia (Bertram, 1988; Chardonnet, 2007; Correll & Houston, 1999; Gilbert et al., 2018), Morocco (Müller & Engel, 2004; SCF, 2020) and Chad (SCF, 2020). Re‐establishing addax in former range countries began in Tunisia in 1985 with an introduction to Bou Hedma National Park (NP; with the entire population later transferred to the Haddej area within Bou Hedma NP) outside the indigenous range of the addax but formed the first step to returning the species to a country that it had been absent from since 1932 (Gilbert et al., 2018). This population formed the basis for two reintroductions to Jbil and Senghar‐Jabbes NPs, within their indigenous range, in the mid‐2000s (Figure 1b). A single augmentation was carried out to Jbil NP in 2007 with addax carefully selected from the EEP and SSP to maximize genetic diversity. Although populations sizes of these population are monitored, there has not been an evaluation of their genetics.

With addax on the brink of extinction in the wild and its future largely reliant on the sustainability of ex situ and reintroduced populations, we undertook an evaluation of the genetic diversity of the global addax population to provide crucial information for the management of the species across the management spectrum (Hoffmann et al., 2015). Biodiversity in the Sahelo‐Saharan region is understudied (Durant et al., 2014), and this evaluation of the genetic diversity of one of its most iconic species adds considerable knowledge to the area. We aim to provide the first global assessment of addax genetics, including samples from wild, ex situ populations (Europe, North America and UAE), and the reintroduced Tunisian population for use in conservation management decision‐making.

## 2 MATERIALS AND METHODS

### 2.1 Sampling and DNA extraction

Faecal samples were collected from wild addax during field surveys in the Tin Toumma region, Niger (2012–2017, *n* = 29) and Chad (2001, *n* = 3). Samples from ex situ addax populations, including hair, tissue and blood (EDTA), were collected opportunistically during routine veterinary procedures from EEP (*n =* 56) and SSP (*n* = 43) institutions in Europe and North America, respectively, and Al Ain Zoo (*n* = 62) and the Environment Agency—Abu Dhabi (EAD; *n* = 63) in the United Arab Emirates. Samples were collected from 102 of 108 censused addax across the three reintroduced populations in Tunisia using biopsy darts. Further sampling details are shown in Table 1 and detailed in File S1.

**TABLE 1:** Samples from global addax populations included in this study.

Sample origin	Population code	Sample collection dates	Population size estimate at time of sampling	Sample type	Number samples	Number successful at mtDNA	Number successful at nuclear DNA
Tunisia	Tunisia		108		104	102	95
Haddej	Haddej	2017	59	Tissue	55	55	51
Jbil NP	JNP	2017	16	Tissue	16	15	14
Senghar‐Jabbes NP	SJNP	2017	33	Tissue	33	32	30
UAE					137	125	105
Al Ain Zoo	AAZ	2010–2019	~280	Blood	67	62	39
Environment Agency—Abu Dhabi	EAD	2018–2019	~75	Blood	70	63	66
Europe (EEP)			~230		65	56	34^a^
8 EAZA institutions	EEP	2012–2019		Hair	19	18	NA
				Blood/Tissue	44	36	34
				GenBank	2	2	NA
USA (SSP)			~225		43	43	42^b^
7 AZA institutions	SSP	2010–2018		Blood/Tissue	43	43	42
Wild			<100		37^d^	32	0
Niger (Termit region)	Niger	2012–2017		Faeces^c^	34	29	NA
Chad (Egeui, now extinct)	Chad	2010		Faeces^c^	3	3	NA
Total					386	358	276

*Note*: Population sizes frequently change, and estimates are provided as a guide only. NA indicates the samples were of insufficient quality and were not included in nuclear DNA analyses.

^a^ Molecular species identification has been carried out and all samples confirmed as addax.

^b^ From three institutions.

^c^ From six institutions.

^d^ Each sample is assumed to originate from a different individual (*n* = 37). Samples with different mitochondrial lineages cannot originate from the same individual and so an absolute minimum of 11 individuals are present but more likely a minimum of 16 individuals were sampled after accounting for sampling timeframes.

DNA was extracted from blood and tissue samples using either of the DNeasy Blood & Tissue kit (QIAGEN), QuickGene DNA whole blood kit S or tissue kit S (FUJIFILM Wako Chemicals Europe GmbH) following the manufacturers' protocols. Samples from AZA institutions were additionally treated with RNase A as per Qiagen standard protocols for the tissue type. Faecal samples were extracted using the QIAmp DNA Stool Mini Kit (QIAGEN) or the Isohelix Xtreme DNA Kit (Cell Projects Ltd) by surface swabbing following the protocol described in Werhahn et al. (2017).

### 2.2 Mitochondrial DNA analyses

All faecal samples were molecularly identified as addax through sequencing of cytochrome b following Verma and Singh (2002). A 723‐bp fragment of the control region was amplified from all sample types using custom‐designed primers AdnewF (5′–GCTATAGCCCCACTATCAAC) and AdnewR (5′–GCGGGTTGCTGGTTTCACGC). PCR products were sequenced in both directions on an ABI 3130XL genetic analyser—see File S1 for additional details.

The resulting sequences were aligned along with 11 published control region sequences for addax (GenBank accessions: JN632591 (Hassanin et al., 2012), MZ474955–MZ475965 (Hempel et al., 2021)). Unique haplotypes were identified using the R package haplotypes (Aktas, 2020). Alphabetical nomenclature was assigned to haplotypes in contemporary samples, and existing GenBank accession numbers used for haplotypes unique to museum samples. A 76‐bp indel was detected in some haplotypes, and the impact of indel re‐coding methods was assessed (see File S1), before selecting the simple index coding method (Simmons & Ochoterena, 2000). The R package pegas (Paradis, 2010) was used to estimate nucleotide and haplotype diversity, deviation from neutrality using Tajima's *D* with significance estimated under a beta distribution (Paradis, 2020) with Bonferroni correction and statistical parsimony (TCS) haplotype networks (Templeton et al., 1992).

### 2.3 Nuclear DNA analyses

A double‐digest restriction‐site associated DNA (ddRAD) library was prepared from samples submitted as blood or tissue following a modified Peterson et al. (2012) protocol (Brown et al., 2016; Manousaki et al., 2016; see File S1). Samples from hair and faeces were degraded and therefore of insufficient quality for ddRAD analyses.

Briefly, DNA was fragmented using the restriction enzymes SphI and SbfI, and a unique pair of 5‐ or 7‐bp barcodes was ligated to each sample. The pooled library was then size selected between 400 and 700 bp using gel electrophoretic extraction, prior to PCR amplification to incorporate adapter sequences. Samples were sequenced across eight libraries, and positive controls were used to confirm repeatability within and between libraries (Leigh et al., 2018). Each library was sequenced (150 bp, paired‐end) on a full lane of an Illumina HiSeq.

stacks v2.52 (Catchen et al., 2013; Rochette et al., 2019) was used to demultiplex the raw data. Reads were aligned using BWA (Li & Durbin, 2009) to the reference genome of the closely related scimitar‐horned oryx (*Oryx dammah* assembly version 1.1, GCF_014754425.2; Humble et al., 2020). Preliminary analyses demonstrated highly concordant results using de novo SNP calling with stacks (using m = 6, M = 2, *n* = 2 after parameterization), which is in line with other studies, suggesting that genomes of closely related species are suitable for SNP discovery in a conservation context (Galla et al., 2019; Samaha et al., 2021). SNPs were called using stacks within a custom snakemake pipeline (Köster & Rahmann, 2012). Details of SNP filtering are included in File S1, but briefly SNPs on autosomal chromosomes with a minimum genotyping rate of 95% and linkage disequilibrium (LD) *R* ^2^ < 0.5 were retained—known as the relaxed LD SNP set (1704 SNPs). A stringent LD SNP set was generated for analyses requiring minimum LD (admixture) for which only SNPs with *R* ^2^ < 0.2 were retained (1073 SNPs).

Population structure was initially assessed using principal component analysis performed within the R package adegenet (Jombart, 2008). The package admixture (Alexander et al., 2009) was then used with the stringent LD SNP set, assessing *K* 2–8 with a 10‐fold cross‐validation with 200 bootstrap resampling runs to estimate the standard errors. Lastly, structure was used to provide a Bayesian estimation of population structure (Pritchard et al., 2000), implemented using the package Parallelstructure (Besnier & Glover, 2013). As structure assigns clusters by maximizing HWE, which is frequently violated in managed populations, and related individuals exist in the dataset which enhances deviation from HWE due to shared haplotypes, we ran structure using the relaxed LD SNP set as well as the stringent LD SNP set. The admixture model was run using a burn‐in of 50,000 followed by 100,000 repetitions for each of *K* 2–10 with 10 repetitions at each value of *K* (with all other parameters as default). Results for both admixture and structure were visualized using the pophelper package for R (Francis, 2017) and CLUMPP (Jakobsson & Rosenberg, 2007). We also performed the admixture and structure analyses without the Tunisian metapopulation to assess whether ancestry estimation was affected by the large number of samples from this divergent reintroduced population.

Observed heterozygosity (*H*_O_), expected heterozygosity within populations (*H*_S_) and the fixation index (*F*_IS_) were calculated using the hierfstat package (Goudet, 2005). Standardized multilocus heterozygosity (sMLH) was calculated using the inbreedr package (Stoffel et al., 2016) to generate an estimate of inbreeding, which is unaffected by allele frequencies. In addition, F and Fhat3, which are affected by allele frequency estimates, were calculated using plink (Chang et al., 2015), and overall levels of relatedness within the population were assessed using the KING‐robust kinship estimates calculated in king (Manichaikul et al., 2010). Allelic richness (Ar) and private allelic richness (pAr) were calculated using adze (Szpiech et al., 2008), standardized to *N* = 14 (the smallest sample size of Jbil NP). Pairwise *F*_ST_ was calculated using the dartr package for R (Gruber et al., 2018) implementing the Weir and Cockerham measure (Weir & Cockerham, 1984), with 1000 bootstraps used to estimate 95% confidence intervals. AMOVA was calculated using the R package poppr (Kamvar et al., 2014), and significance was tested using the randomization procedure implemented within the R package ade4 (Dray & Dufour, 2007) using 1000 repeats. All analyses were carried out within R v4.0.3 (R Core Team, 2020).

## 3 RESULTS

### 3.1 Mitochondrial DNA

A 723‐bp fragment of the mitochondrial control region was obtained from 359 samples, and we identified a total of 25 haplotypes (Table S1, Figure S1), 18 identified within contemporary samples. We identified nine haplotypes within the 29 faecal samples collected from Niger between 2012 and 2017, and two additional haplotypes from three faecal samples collected from Chad in 2001 (Table S1), resulting in a minimum of 11 individuals represented within these samples. We identified eight haplotypes from 324 individuals and an additional two GenBank sequences across ex situ populations and the reintroduced population in Tunisia. Only haplotype *N* was detected in the wild and a contemporary population (Tunisia; Table S1). Of the eight haplotypes identified by Hempel et al. (2021) from museum specimens, a single haplotype (M) was also detected in two samples from the presumed extinct population in Chad. Mean haplotype diversity across all haplotypes was 0.799 and mean nucleotide diversity was 0.0047, and diversity estimates are higher for wild populations than ex situ and reintroduced (Table 2).

**TABLE 2:** mtDNA diversity between wild and managed populations at 723 bp of d‐loop.

Grouping	Populations	*N* samples	*N* haplotypes	Nucleotide diversity	Haplotype diversity	Tajima's *D*
Wild (pre‐1930)^a^	Libya, Sudan, Tunisia, Western Sahara	9	8	0.0113	0.9722	−0.305
Wild (contemporary)	Niger (2012–2017)	29	9	0.0137	0.8867	1.391
	Chad (2001)	3	2	0.0083	0.6667	NA^b^
Managed populations (Ex situ + reintroduced)	AAZ	62	4	0.0037	0.6409	−0.795
	EAD	63	5	0.0036	0.6569	−0.968
	EEP	56	3	0.0021	0.5864	−0.619
	SSP	43	4	0.0032	0.6877	−1.009
	Tunisia	102	6	0.0013	0.5636	−1.902*
	Ex situ combined	326	8	0.0034	0.7604	−0.956
Tunisian metapopulation	Haddej	55	4	0.0010	0.5556	−1.681
	JNP	15	6	0.0030	0.8667	−2.210*
	SJNP	32	2	0.0004	0.2258	−0.138

*Note*: Significance of Tajima's *D* is indicated by * and significance after Bonferroni correction is indicated by bold. In all cases, estimates for Tunisia summarise all three national parks, which are also shown independently (shaded in grey).

^a^ Museum sequences from Hempel et al. (2021).

^b^ Tajima's *D* cannot be calculated for a sample size of 3.

**FIGURE 2:**
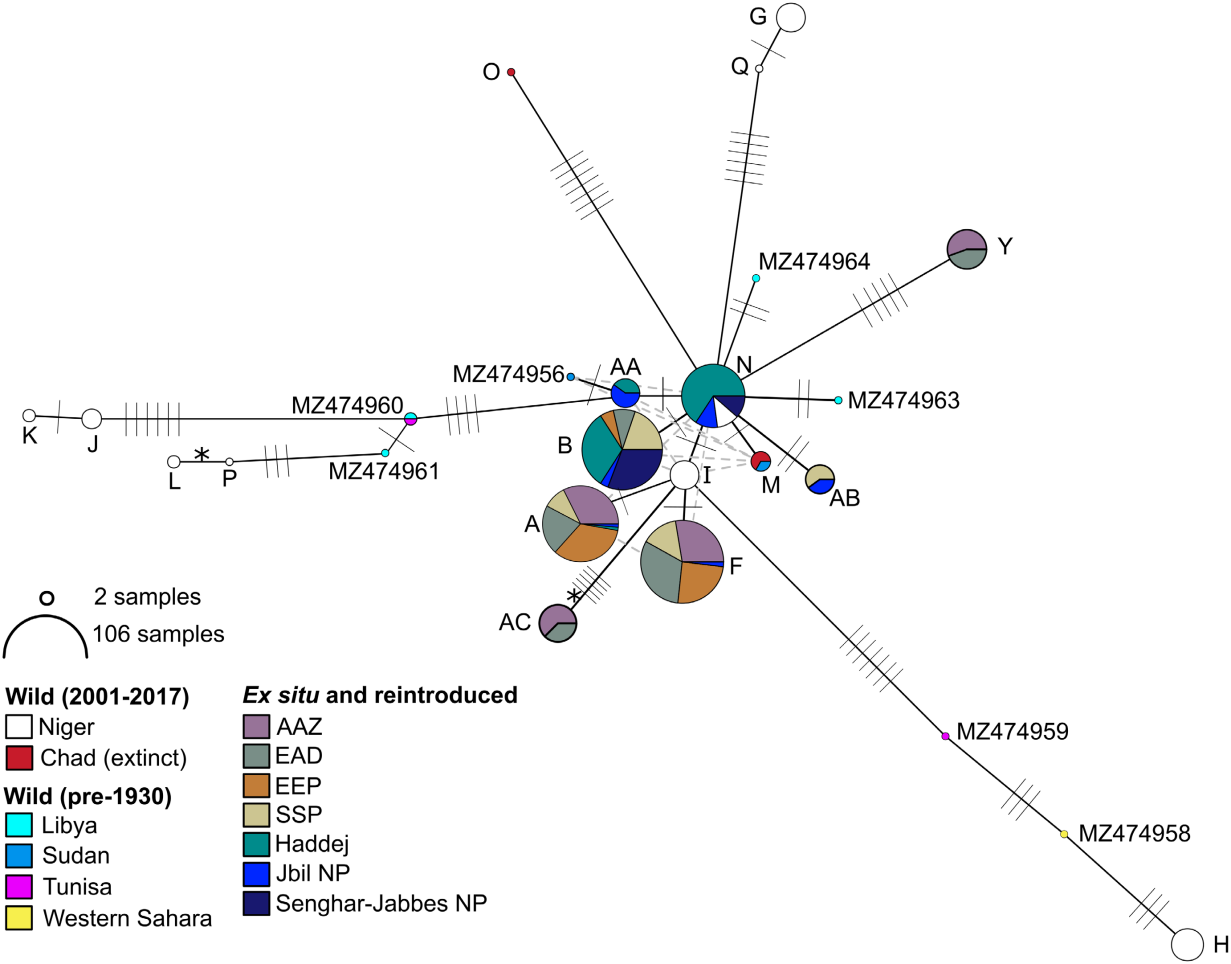
Network of addax mtDNA control region haplotypes. Circles represent haplotypes, with most likely evolutionary relationships indicated by solid black lines and alternative likely relationships shown by dashed grey lines. Mutational steps are shown by hashes. The 76‐bp indel was recoded as a single base pair 5th state and is indicated by *; all other indels were excluded. Haplotype circles are coloured according to populations as shown, with circle size indicating sample size (log scale) as indicated. Haplotype nomenclature follows Table S1. Wild (pre‐1930) haplotypes are from Hempel et al. (2021).

Evolutionary relationships between the observed haplotypes are shown in Figure 2. The eight haplotypes from ex situ and reintroduced populations form a tight central cluster, with haplotype Y being the most divergent (five mutational steps from the nearest haplotype). Haplotypes from wild populations were more diverse. Average nucleotide diversity was also greater in wild haplotypes (pi = 0.014) than in ex situ/reintroduced haplotypes (pi = 0.003).

### 3.2 Nuclear DNA

We analysed genome‐wide nuclear SNP markers for 276 individuals. Across eight ddRAD libraries, 322 samples were sequenced representing 276 individuals, including 18 samples sequenced between two and six times each as positive controls. On average, 6,226,520 reads were sequenced per sample (range: 14,475–29,460,298), with an average of 5,751,874 (91.2%) reads that were mapped against the scimitar‐horned oryx genome (range: 39.6%–97.1%). We identified a total of 24,395 SNPs, and after filtering, the relaxed LD SNP set retained 1704 SNPs with a genotyping rate of 95% across individuals.

### 3.3 Global population structure

**FIGURE 3:**
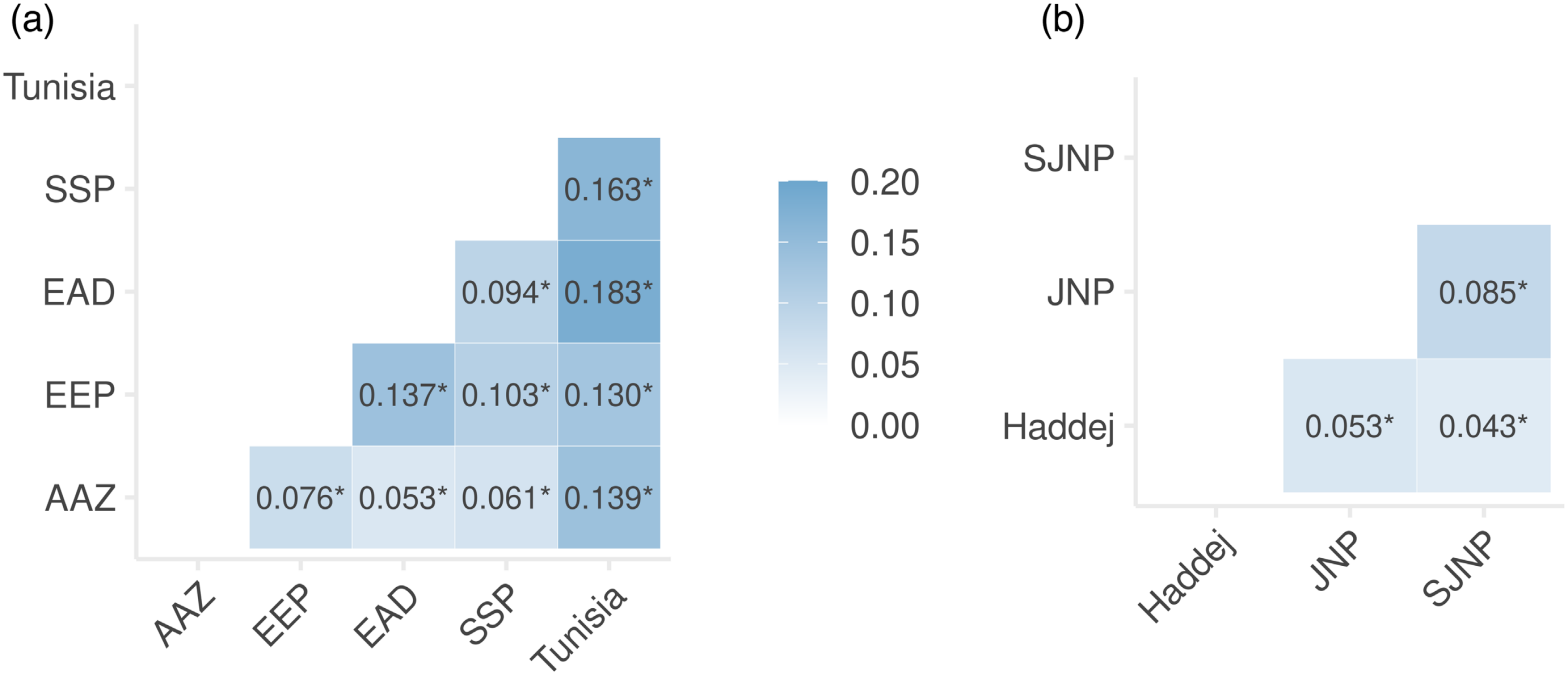
Pairwise *F*_ST_ estimates for the five primary ex situ populations (a) and for the three Tunisian populations (b). All pairwise *F*_ST_ estimates were significant (as indicated by *).

**FIGURE 4:**
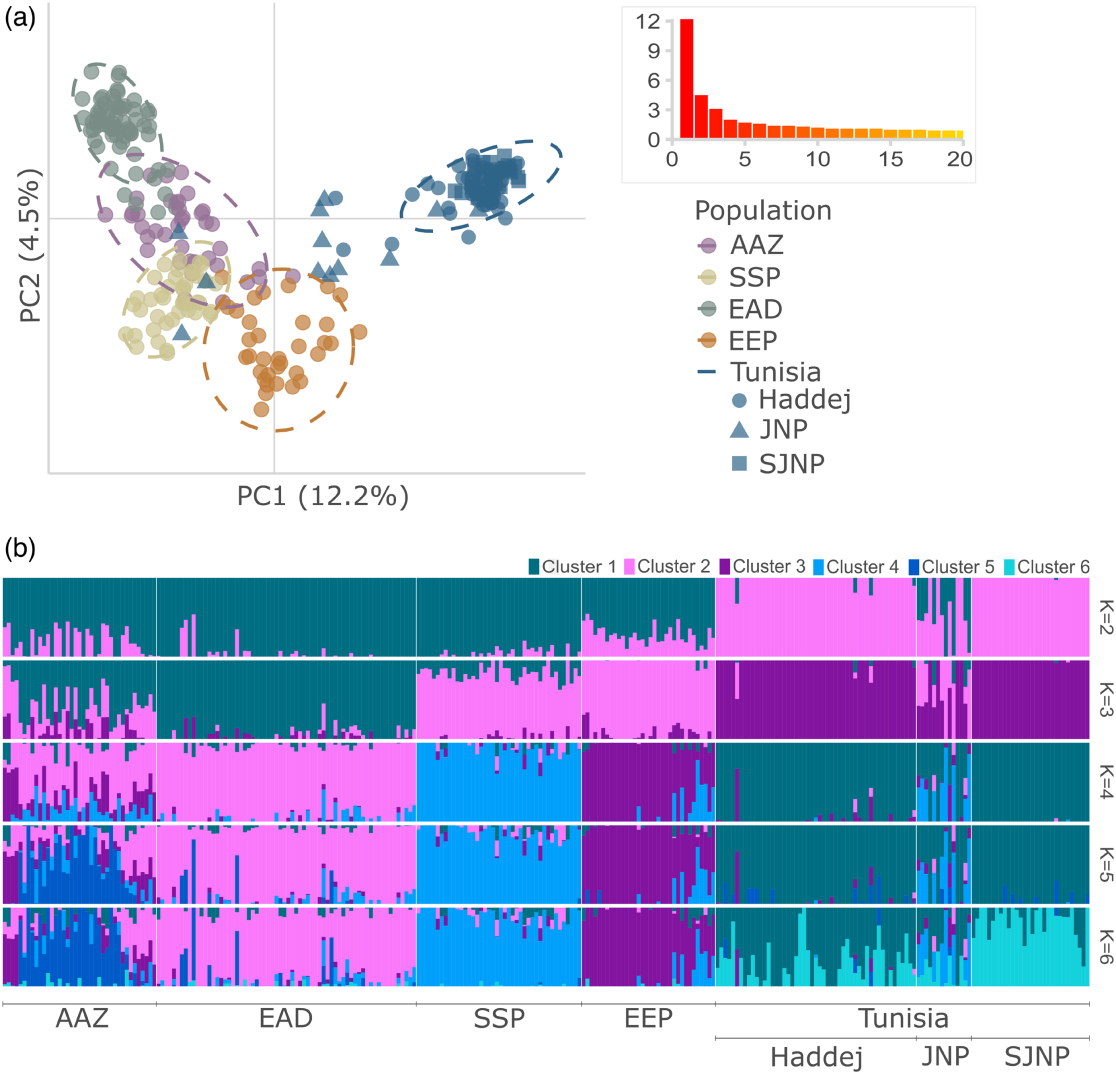
Visualization of population structure in the ex situ and reintroduced addax populations using two methods. (a) PCA using 1704 SNPs in the managed addax populations, showing PC1 and PC2 (percentage of variation explained shown in brackets). The Tunisian metapopulations are represented by different shapes, as shown in the legend. Inset shows the first 20 eigenvalues. (b) Admixture results using 1073 SNPs filtered to minimize linkage disequilibrium, showing, for each individual, the proportion of genetic membership to each ancestry cluster for the most informative number of clusters (*K* = 2 to *K* = 6). *K* = 5 was the best supported model.

AMOVA showed that over 83.9% of the variation is partitioned within individuals (Phi = 0.161, *p* = 0.001, Table S2). There was evidence of variation at the population level, with 12.9% of the variation distributed amongst the five primary ex situ populations (Phi = 0.129, *p* = 0.001). *F*_ST_ ranged between 0.053 (AAZ–EAD) and 0.183 (EAD–Tunisia), shown in Figure 3a, with all measures significant (*p* < 0.001). *F*_ST_ estimates within Tunisia (Figure 3b) show that divergence is greatest between Jbil NP and Senghar‐Jabbes NP (*F*_ST_ = 0.085 ± 0.009 95% CI). *F*_ST_ estimates within Tunisia were lower than comparisons between the Tunisian metapopulation as a whole and other global populations, though pairwise comparisons including Jbil NP (Jbil NP–Haddej *F*_ST_ = 0.053; Jbil NP–Senghar‐Jabbes NP *F*_ST_ = 0.085) were similar or greater than estimates between some global populations (Figure 4a, AAZ–EAD *F*_ST_ = 0.053).

We carried out an initial assessment of population structure using PCA, shown in Figure 4a. Principal component (PC) 1 accounted for the greatest proportion of variation within the dataset (11.3%), which primarily separates the Tunisian metapopulation from other global populations. The remaining four ex situ populations formed continuums along PC1 and PC2, with none forming independent cluster. This pattern was supported by subsequent PCs (Figure S2).

To explore the population structure at a finer scale, we used admixture to investigate individual ancestry using 1073 SNPs filtered to minimize linkage disequilibrium. Cross‐validation suggested that five clusters were the most supported model (Figure S3), and results of *K* = 2 to *K* = 6 are shown in Figure 4b. At all estimates of *K*, Tunisia remained a distinct cluster. The EEP and SSP largely formed an independent cluster at *K* = 3 and become distinct from one another at *K* = 4, although the EEP population included several individuals more similar to the SSP population than to other EEP addax. The two Arabian populations (AAZ and EAD) show ancestry similarly differentiated from other populations, but with varying levels of admixture at all estimates of *K*. The AAZ is composed of at least two clusters with varying levels of admixture from *K* = 4 onwards. After excluding the Tunisian population, there was no substantial difference in the resulting population structure (Figure S4). Additional analyses using structure showed broadly similar results (Figures S5 and S6) with higher levels of admixture depending upon the degree of filtering to minimize linkage disequilibrium.

Within Tunisia, Jbil NP was found by PCA to include several individuals that did not cluster closely with addax from Haddej or Senghar‐Jabbes NP and three individuals clustering with the SSP (Figure 4a), one of which was a surviving founder translocated from the SSP to Jbil NP in 2007. admixture analyses showed evidence of high levels of admixture within the Jbil NP addax (Figure 4b), with genetic signatures from the SSP, the EEP and the wider Tunisian population at all values of *K*.

### 3.4 Genetic diversity within populations

**FIGURE 5:**
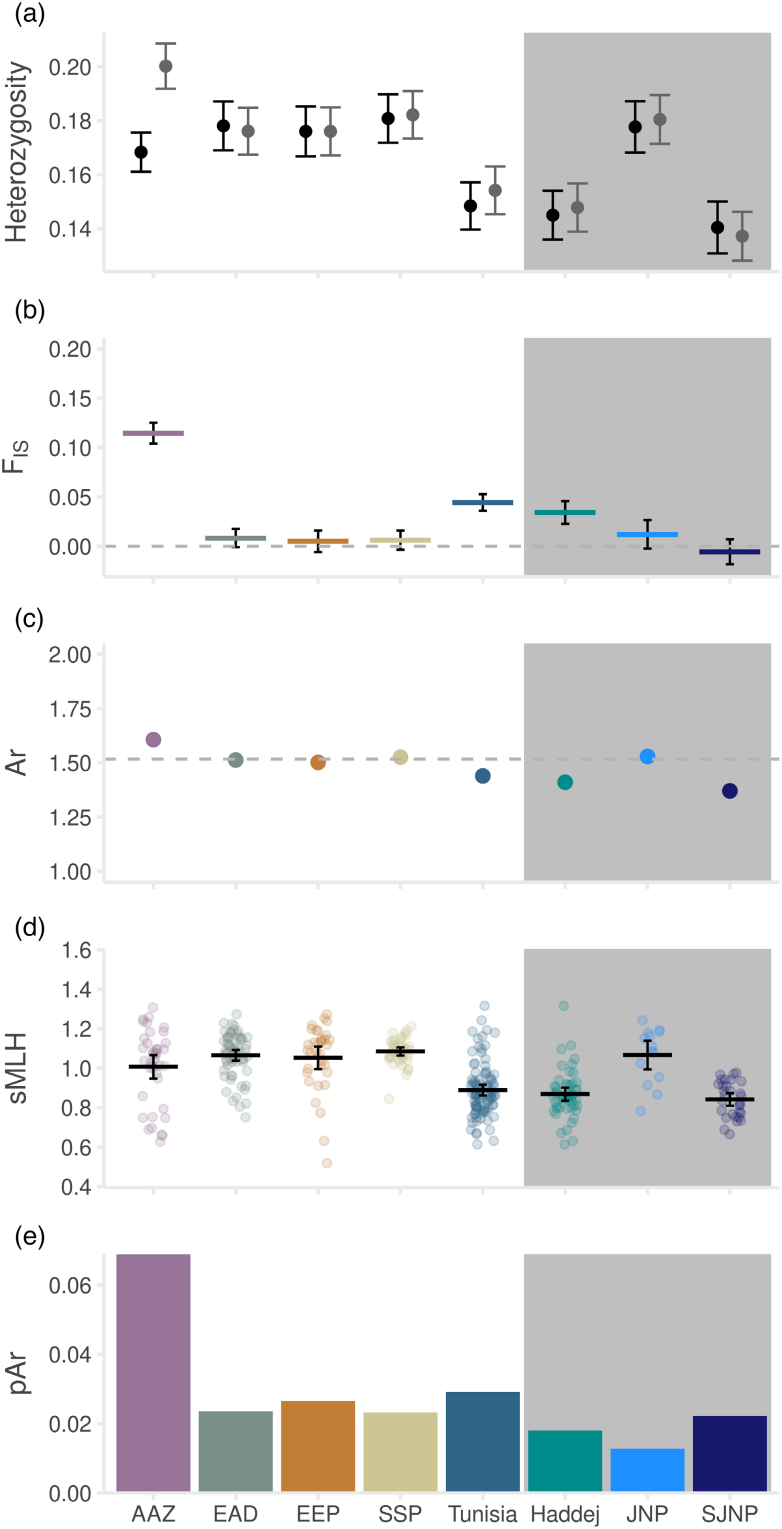
Genetic diversity measures in the ex situ and reintroduced populations. (a) Mean observed heterozygosity (black points) and expected heterozygosity (grey points) for each population, with 95% confidence limits (black and grey bars, respectively). (b) Mean *F*_IS_ (coloured bars) within each population and 95% confidence limits (black error bars). (c) Mean allelic richness (Ar) (points) standardized to *N* = 14, with 95% confidence limits (black error bars), with the global average indicated by the horizontal dashed line. (d) Individual standardized multilocus heterozygosity (sMLH). (e) Private allelic richness (pAr) standardized to *N* = 14. In all cases, estimates for Tunisia summarize all three national parks, which are also shown independently (shaded in grey).

Estimates of genetic diversity were similar across the EAD, EEP and SSP populations, but deviations from Hardy–Weinberg expectations were detected in both AAZ and Tunisia (Figure 5, Table S3). For AAZ, observed heterozygosity was significantly lower than expected heterozygosity (*H*_O_ = 0.168 [95% CI 0.161–0.176], *H*_S_ = 0.200 [95% CI 0.192–0.209]), and *F*_IS_ was raised (*F*_IS_ = 0.115, [95% CI 0.104–0.125]). However, mean sMLH for AAZ was in line with the other ex situ populations despite high variation in individual sMLH estimates, and pairwise kinship (KING‐robust) estimates were very low (mean KING = −0.2).

Within Tunisia, only *F*_IS_ was raised (*F*_IS_ = 0.044 [95% CI 0.036–0.053]) and this was largely driven by Haddej (Figure 5b, Table S3). sMLH (Figure 3c) was also reduced in Tunisia as a whole, which seems to be driven by the lower individual diversity in Haddej and SJNP than in JNP. Estimates of inbreeding using F and Fhat3 follow a similar pattern (Figure S7A,B), and low levels of kinship (Figure S7C) suggest that this pattern is not driven by an excess of related individuals within our dataset.

## 4 DISCUSSION

The data presented here provide a genetic baseline that is needed to underpin sound international population management of Critically Endangered addax across the in situ wild population and ex situ populations that are managed to varying extents. The resolution of genetic data is intrinsically tied to sample quality, which in turn is often tightly linked to the type of source population. Challenging field conditions meant that faecal samples were the only sample type available from the wild, including the now extinct population in Chad and the remnant population in Niger. The mitochondrial data generated from these samples provide a first insight into the genetic status of wild addax, as well as a vital overview between wild, reintroduced and ex situ populations. Wild faecal DNA quality was insufficient for ddRAD analysis, but SNP data generated from the higher quality blood and tissue samples collected from ex situ populations provide data to guide population management decisions at an institutional, regional and global level.

### 4.1 mtDNA diversity is greater in situ than ex situ

We detected nine mtDNA haplotypes from 29 faecal samples collected in Niger between 2012 and 2017, and we detected two additional haplotypes from three samples collected in 2001 in Chad. Individual identification was not possible using mtDNA, though these 33 wild samples must therefore represent a minimum of 11 unique individuals. By the early 2000s, the wild addax population was estimated to be fewer than 300 individuals in two populations in Niger and Chad (Newby, 2013; Newby & Wacher, 2008). By 2016, this estimate was revised to 30–90 individuals (IUCN SSC Antelope Specialist Group, 2016), and the population in Chad is now thought to be extinct (IUCN SSC Antelope Specialist Group, 2020).

Previously analysed mitogenomes revealed weak phylogeographic structure across the historic range of addax (Hempel et al., 2021), and our analyses of the control region in contemporary samples continue to support this lack of geographic signature (Figure 2). Unsampled haplotypes, such as those now extinct, impact the accuracy of haplotype network reconstruction (Paradis, 2018), and we struggled to reconstruct evolutionary relationships amongst haplotypes carrying the 76‐bp indel. Nevertheless, the networks generated from mitogenomes (Hempel et al., 2021) and the single control region locus analysed here were largely congruent. The lack of phylogeographic signal may reflect high geneflow as a result of the perpetually nomadic nature of addax (Hempel et al., 2021), which once ranged long distances to find food and water in the Sahel and Sahara and gathered seasonally in herds of hundreds of individuals (Beudels‐Jamar et al., 2005).

mtDNA diversity within the ex situ populations and those in Tunisia, which were sourced from them, (326 individuals across 18 institutions) was reduced compared with the contemporary wild population (Figure 2). Only eight haplotypes were detected, and both nucleotide diversity (wild 0.014, ex situ 0.003) and haplotype diversity (wild 0.905, ex situ 0.761) were lower (Table 2). Museum samples from Hempel et al. (2021) revealed additional diversity (Table 2, Figure 2), and it is apparent that the ex situ population only captured a small subset of the historical diversity within addax. Whilst the TCS network reveals uncertainty surrounding the precise placement of several haplotypes from ex situ populations, they remain tightly clustered compared with contemporary and museum haplotypes from the wild (Figure 2). mtDNA diversity retained in ex situ addax populations is lower than closely related arid land Hippotragini species, which were declared Extinct in the Wild, the scimitar‐horned oryx (*Oryx dammah*) with 43 control region haplotypes (Ogden et al., 2020) and the Arabian oryx (*Oryx leucoryx*) with 11 control region haplotypes (Ochoa et al., 2016).

#### 4.1.1 Nuclear DNA provides insights into genetic diversity within and relationships between ex situ populations

ddRAD analyses revealed population structure amongst the ex situ and Tunisian addax (Figure 3, Table S2), with distinct clusters corresponding to the five primary populations assessed (Figures 4 and 5). Estimates of population and individual diversity are similar amongst the EEP, SSP and EAD, despite higher intensity management within the EEP and SSP compared with the EAD. The history of the EAD herd is not well documented. The genetic evidence suggests similarities between the EAD and SSP (Figure 4 and Figure S6) that may indicate recent shared ancestry, as well as the presence of several admixed individuals indicating geneflow into the population. The population at AAZ, however, has an excess of homozygotes (Figure 4a) and raised *F*_IS_ (Figure 4b), but no substantial difference in mean sMLH compared with other populations (Figure 4e). We suspect that a Wahlund effect, a heterozygote deficit caused by the presence of subpopulations (Allendorf & Luikart, 2006), may have contributed to this pattern, caused by a recent import of addax from at least two other, unsampled UAE institutions (M.Y. Al Faqeer, pers. Comm.). However, variance in sMLH is high within AAZ samples (Figure 4e), suggesting that some individuals may indeed have raised inbreeding levels. This recent geneflow into AAZ could have contributed to the population's raised Ar and pAr and mixing amongst subpopulations should act to reduce inbreeding in subsequent generations.

#### 4.1.2 Semimanaged populations in Tunisia provide a window on the genetic outcomes of reintroduction

The Tunisian metapopulation was genetically distinct across all our analyses despite being entirely founded approximately 6–7 generations ago from EEP and SSP individuals. This is indicative of a bottleneck effect at the founding of this population with rapid genetic drift. The pattern is, however, driven by Haddej and Senghar‐Jabbes NP, with Jbil NP much more similar to the EEP and SSP source populations. Although samples from the Tunisian founders were not collected for genetic analysis, the contemporary partitioning of diversity throughout the metapopulation provides an indication of the success of the differing stepping‐stone and augmentation strategies used.

The initial 1980s Tunisian reintroduced population was founded with only 14 individuals from two institutions with high relatedness amongst founders (Figure S8), and Haddej and Senghar‐Jabbes NP addax are descended from only these founders. Low levels of diversity and heterozygosity in these populations are in line with predictions that genetic diversity declines are more pronounced in smaller populations (Frankham, 1996) and reserves (Heller et al., 2010), and following founder events, particularly serial and stepping‐stone founder events (Excoffier et al., 2009; Slatkin & Excoffier, 2012). An augmentation of Jbil NP using 13 genetically diverse individuals selected from the EEP and SSP using pedigree analyses is likely acting to genetically rescue this population. Genetic diversity within Jbil NP is similar to its source populations, with higher mitochondrial and nuclear diversity than its counter parts in Haddej and Senghar‐Jabbes NP despite its smaller population size (Table 1). However, there has been insufficient time since augmentation to assess whether this increased diversity translates to increased population fitness and therefore genetic rescue (Whiteley et al., 2015), nor is it clear if reduced genetic diversity has yet resulted in increased genetic load, inbreeding depression and reduced fitness in the nonaugmented populations. Assessments of genetic load are becoming increasingly plausible with whole‐genome sequencing (Dussex et al., 2021; Humble et al., 2022; Khan et al., 2021; von Seth et al., 2021). Inbreeding depression and fitness are notoriously difficult to assess in species of conservation concern (but see Trask et al., 2021), requiring intensive monitoring to generate the necessary life‐history data.

#### 4.1.3 Conservation management implications for addax

This work has demonstrated the critical importance of the remaining wild population of addax within Niger as a genetic reservoir as, despite the diminishing size of this population, it retains notably greater genetic diversity than the substantially larger global ex situ and reintroduced populations combined. The combined SSP and EEP ex situ populations (which share a high proportion of founders) are thought to descend from a maximum of 42 individuals (Enright, 2019), above the general rule of 20–30 founders (Foose, 1983; Foose et al., 1986; Lacy, 1987). However, our data suggest they do not represent the recommended 20 genetically diverse individuals needed to capture 97.5% of genetic variation (Lacy, 1989), likely due to limited numbers and localities of founder catch‐ups. Whilst addax ex situ populations have, and will undoubtedly continue, to play a pivotal role in preventing extinction, conservation efforts must focus on protecting the last wild population.

Ultimately, the ability of a population to adapt to its environment over the long term is determined by the amount of diversity in the form of allelic variation (Allendorf, 1986; Caballero & García‐Dorado, 2013). Both the mtDNA and nuclear data presented here indicate that the Tunisian metapopulation is genetically depauperate compared with the ex situ population as a whole and to individual ex situ populations. Although the Tunisian metapopulation has remained managed to date, the ultimate aim is to establish a wild, self‐sustaining population within the historic range in southern Tunisia. Despite the limited genetic diversity within Tunisia's Haddej and Senghar‐Jabbes NP, these populations have persisted under management. Species and populations have recovered from the brink of extinction with remarkably few founders (Groombridge et al., 2012; Kennedy et al., 2014; Santymire et al., 2014), but substantial reductions in genetic diversity are inevitable. Severe bottlenecks and associated reductions in genetic diversity have been widely shown to have long‐lasting effects on fitness and population persistence (Bouzat, 2010; Frankham, 2005; Ralls et al., 2020). This Tunisian addax case study has illustrated the need for both careful selection of founders for reintroduction and ongoing genetic monitoring, and it further illustrates that collection of genetic samples from the outset should be a critical part of any translocation (Brockett et al., 2022), ideally with subsequent submission to a biobank for long‐term preservation. Lastly, genetic augmentation through multiple releases may be needed to increase diversity and counteract drift (Dlugosch & Parker, 2008).

Given the perilous state of wild populations of Sahelo‐Saharan megafauna, conservation translocations are an increasingly critical tool in securing populations within their indigenous range, and reintroductions have already proven successful for antelope in North Africa. Levels of genetic diversity within ex situ populations are relatively low, with limited genomic diversity and structure, and the Tunisian reintroduction highlights that poor founder selection without subsequent genetic management can lead to concerningly low levels of genetic diversity. Therefore, maximizing diversity within future addax reintroductions by careful selection of founders from multiple source populations is advised. Managing the partitioned genetic diversity found within ex situ addax populations to preserve genetic variants and minimize impacts of localized inbreeding must now be a key objective of integrated conservation planning for the species, and it is urgent that both in situ and ex situ managers and stakeholders cooperate in a global conservation management plan to ensure maximal retention of genetic diversity.

#### 4.1.4 Use of genetic data to support integrated conservation planning

Integrating management and conservation planning across in situ and ex situ populations, for example under a ‘One Plan Approach’ (Byers et al., 2013; Traylor‐Holzer et al., 2019), is increasingly important for species requiring conservation. This genetic assessment of wild, ex situ and reintroduced populations of addax highlights the value of generating such genetic information for baseline conservation planning, which is likely echoed across many species of conservation concern. Understanding how genetic diversity is partitioned can assist with directing conservation action to particular populations, for example towards wild addax using the data generated here (IUCN SSC Antelope Specialist Group, 2020), improve pedigree‐based management of ex situ populations (Galla et al., 2022; Hogg et al., 2017; Ivy et al., 2016), the relevance of group management strategies to integrate populations with varying intensities of management (Gooley et al., 2022), develop metapopulation management approaches (Gooley et al., 2020, 2022; Magliolo et al., 2022; McLennan et al., 2018; Wright et al., 2021) and identify source populations and optimal strategies for reintroductions (Bozzuto et al., 2017; Jackson et al., 2022; Ochoa et al., 2016; Ogden et al., 2020). Such approaches are of particular interest for addax conservation and can be explored using the baseline data generated here.

Our experience has highlighted that the availability of appropriate samples for genetic analyses remains one of the greatest challenges with generating such data for species on the brink of extinction. Analyses of wild populations are frequently subject to the well‐documented challenge of applying modern molecular methods to degraded DNA (but see de Flamingh et al., 2022; Fontsere et al., 2021; Taylor et al., 2021). However, the availability of high‐quality samples from some ex situ populations was also limited. There is a clear need to improve genetic resources for addax to facilitate conservation management, and biobanking, cell line generation and reproductive technologies must be prioritized for this species. Improved availability of high‐quality genetic samples from key ex situ populations is vital for developing the data to enable integrated population management. Given the global nature of these ex situ populations and challenges associated with international translocations, movement of germ cells may prove crucial to establishing effective geneflow. Such resources may prove invaluable to the conservation of addax in the long term, but, nevertheless, the need for in situ conservation action and improved global population management of ex situ populations is immediate.

## FUNDING INFORMATION

Funding for this research was provided by the institutions listed in author affiliations: Al Ain Zoo, the Environment Agency—Abu Dhabi, Marwell Wildlife (UK registered charity number 275433), Royal Zoological Society of Scotland (UK registered charity number SC004064), SaharaConservation and San Diego Zoo Wildlife Alliance. However, the funders did not have any additional role in study design, data collection and analysis, decision to publish, or preparation of the manuscript.

## CONFLICT OF INTEREST

The authors declare that they have no competing interests in relation to this manuscript.

## Supporting information


Appendix S1

Figure S1

Table S1

## Data Availability

Mitochondrial control region haplotypes were submitted to GenBank under accession numbers OP745957–OP745974. The raw, demultiplexed ddRAD data for this study have been deposited in the European Nucleotide Archive (ENA) at EMBL‐EBI under accession number PRJEB53821. Analysis code, including the custom snakemake pipeline for SNP calling, is available on GitHub (github.com/karadicks/Addax_global_genetics), and the filtered genotype data are available on Dryad (10.5061/dryad.dr7sqvb2j). Appropriate access and benefit‐sharing agreements are in place to ensure access, use and benefits of the genetic data produced here for conservation by provider countries.
